# Comparative study of knowledge about oral cancer among undergraduate dental students

**DOI:** 10.1590/S1679-45082016AO3729

**Published:** 2016

**Authors:** Samara Ribeiro da Silva, Yara Juliano, Neil Ferreira Novo, Ilan Weinfeld

**Affiliations:** 1 Universidade de Santo Amaro, São Paulo, Brazil

**Keywords:** Mouth neoplasms, Smoking, Alcoholism, Surveys and questionnaires, Students, dental

## Abstract

**Objective:**

To evaluate and compare the knowledge of dental undergraduate students about oral cancer.

**Methods:**

The students were divided into two groups according to semester attended in the undergraduate course: Group A, the first semester; and Group B, seventh semester. They were asked to answer a questionnaire about epidemiology, risk factors, clinical aspects, therapeutic solutions and oral self-examination. For statistical analysis, the Fisher's exact test, the Cochran's G test and Kendall's concordance test were used, with significance level set at 0.05.

**Results:**

Regarding the prevalent sex, only 8.0% of Group A and 56.0% of Group B judged males as the frequent affected by the disease (p=0.0006). In terms of age, 84.0% of the Group B and 44.0% of the Group A estimated that most cases were diagnosed over 40 years (p=0.0072). Smoking was identified as the major risk factor for 64.0% and 91.6% of Groups A and B, respectively (p=0.0110). On issues related to sex, ethnicity, age, risk factors, self-examination, treatment, professional responsible for treatment and profile of an individual with the disease, the seventh-semester showed significantly higher correct answer percentages than first-semester undergraduates.

**Conclusion:**

There was significant correlation between the right and wrong answers given by first and seventh semester students, making necessary a specific approach directed to their lack of knowledge.

## INTRODUCTION

Generically referred to as oral cancer (mouth cancer), squamous cell carcinoma is the most common malignancy of the oral cavity.^(1–3)^ In dissonance to other neoplasms, such as breast, prostate and skin cancer, this condition is rarely seen in the media; hence, important information regarding risk factors, treatment and self-examination goes unnoticed. The situation described explains the ignorance of the public about the lesion, except by the occurrence of a late diagnosis, invariably resulting in mutilations and serious sequelae, or even interfering with patient survival.

The *Instituto Nacional de Câncer José Alencar Gomes da Silva* (INCA) estimated that 11,140 new cases of oral cancer in men and 4,350 in women will be diagnosed in Brazil, in 2016. Excluding non-melanoma skin tumors, for males, these rates render this type of cancer between the fourth and the seventh most common cancer in Brazil, depending on the geographic region. In women, this condition ranks between the 9^th^ and the 15^th^ most common cancer, also depending on the location in the country.^(4)^ Due to its high incidence, oral cancer is a major public health problem, and attitudes and practices regarding the disease should be reviewed, aiming to reduce its occurrence and encouraging an early diagnosis, including potentially malignant oral lesions, such as leukoplakia, erythroplakia, and actinic cheilitis.^(5)^

Due to the situation described, it is essential that healthcare professionals be able to work at the several levels of disease prevention in their area of expertise. However, dentistry, a science directly involved with oral cancer, plays a key role in its prevention and diagnosis, despite the fact the dental surgeon may not be responsible for the treatment, because this professional is expected to examine and diagnose even the most incipient oral changes.

Preliminarily, undergraduate courses must prepare students and enable them to diagnose any diseases of the stomatognathic system. Thus, an assessment of the academic knowledge at different stages of training, both before and after the semiotic disciplines, is a plausible way to measure the expertise of future professionals, as they encounter different oral changes, especially oral cancer.

Several studies have assessed the views and professional practices regarding the disease, noting that the respondents had a good knowledge of the main risk factors, such as smoking and alcohol consumption.^(1,2)^ However, only 39.0% of dental surgeons and 9.0% of physicians knew how to identify the most common sites in which this type of cancer develops.^(6)^ Interestingly, only a few of them felt prepared to perform the biopsy procedure.^(7)^

Squamous cell carcinoma, in most cases, can be described as an ulcerated lesion with sharp raised and hardened edges with a necrotic center and a hardened base due to the infiltration of the underlying tissue, usually asymptomatic in its early stage and of rapid growth.^(8)^

Thus, in a high risk population for oral cancer, it was observed that three of the four questions about clinical changes that could correlate with the disease (white and reddish lesions in the mouth, ulcers that do not heal, and abnormal cervical lymph nodes) were significantly more likely (p<0.0100) to be correctly answered by a group that was sensitized by prevention campaigns, when compared to a non-sensitized group.^(9)^ Therefore, raising awareness of the population about the disease is an effective method of primary prevention.

For these reasons, the focus of this study was an informative comparative evaluation of several dental students regarding oral cancer, since these future professionals, if duly qualified, will efficiently serve the community. For an effective and appropriate intervention in the social environment, it is important to work at different levels of disease prevention, with particular attention to primary prevention and early diagnosis.

## OBJECTIVE

To evaluate and compare the academic knowledge of the first and seventh semesters of the dentistry undergraduate course regarding the etiology, epidemiology, risk factors, symptoms, clinical features, treatment, prognosis, self-examination and prevention of oral cancer.

## METHODS

This is a cross-sectional study conducted in the city of São Paulo (SP), with students enrolled in the college of dentistry of a university with a traditional teaching-learning model, associated with the following method: cases study presentation to be solved in activities called “seminars”. The students were divided into two groups, according to the semester attended in the undergraduate course at the time of the study, comprising a sample of 25 first-semester students (Group A) and a sample of 25 seventh-semester students (Group B). The population totaled 50 undergraduate students who volunteered to participate in the study by signing the Informed Consent.

The volunteers were asked to answer a questionnaire (Appendix 1) containing 15 multiple-choice questions, with only one correct answer alternative regarding the etiology, epidemiology, risk factors, symptoms, clinical features, treatment, prognosis, self-examination and prevention of oral cancer. The students were instructed to answer it in the period assigned for the application of questionnaires.

For statistical analysis, the following tests were applied: Fisher's exact test, to compare groups A and B regarding the percentages of correct answers; Cochran's G test, to compare the percentages of correct answers to each of the questions posed, applied separately for the above groups; Kendall's concordance test, to demonstrate the concordance in the percentages of correct answers in ascending order, observed in Groups A and B.

The significance level was set at 0.05 or 5%.

This study was previously submitted to Plataforma Brasil and approved by the Research Ethics Committee, according to under number 1.053.885, CAAE: 44507915.1.0000.0081.

## RESULTS

After applying the questionnaires, the answers provided by students of Groups A and B were compared regarding correct and wrong answers for each of the questions, in order to assess, by applying the Fisher's exact test, if there were statistically significant differences between the response rates, as shown in table 1.

**Table 1:** Frequency of right and wrong answers to the questionnaire (significant results in bold) given by first and seventh semester undergraduate dental students

Question	1^st^ Semester			7^th^ Semester			Significance level
	Right	Wrong	% right	Right	Wrong	% right	
Contagious	25	0	100.0	24	1	96.0	p=0.9999
Etiology	20	4	83.3	24	1	96.0	p=0.1895
Prevalent gender	2	23	8.0	14	11	56.0	p=0.0006
Prevalent ethnicity	5	20	20.0	21	4	84.0	p=0.0000
Age group	11	14	44.0	21	4	84.0	p=0.0072
Risk factors	16	9	64.0	22	2	91.6	p=0.0110
Symptoms	15	10	60.0	16	8	66.6	p=0.7698
Clinical features	22	3	88.0	22	3	88.0	p=0.9999
Prognosis	24	1	96	24	1	96.0	p=0.9999
Self-examination	9	16	36.0	20	5	80.0	p=0.0037
Treatment	17	8	68.0	25	0	100.0	p=0.0040
Professional responsible for treatment	4	19	17.4	14	10	58.3	p=0.0065
Patient profile	16	9	64.0	24	1	96.0	p=0.0106
Prevention methods	20	4	83.3	25	0	100.0	p=0.0502
Multidisciplinary intervention	25	0	100.0	25	0	100.0	p=0.9999

Fisher's exact test.

Regarding the epidemiological characteristics, the percentage of correct answers of the seventh-semester undergraduate students were significantly higher than the percentage of correct answers of the first-semester students, as they answered correctly that the lesion was more frequent, mainly, in males, Caucasian, aged over 40 years (Table 1). However, 84.0% of Group A and 36.0% of Group B answered that the disease was equally prevalent in both sexes. About 60.0% of Group A and 12.0% of Group B stated that the lesion affects Black and Caucasian alike. Also, 56.0% of students in Group A and 16.0% of Group B thought that the disease occurred predominantly in individuals aged between 20 and 40 years.

Regarding risk factors, 91.6% of students of the seventh semester and 64.0% of students of the first semester correctly indicated smoking as the main factor in the development of the disease, with significantly higher percentages of correct answers for Group B. Approximately 24.0% of Group A indicated genetic inheritance as the main risk factor and 12.0% of that group declared that there were no established risk factors for this disease.

Similarly, the percentage of correct answers revealed statistically significant differences between the samples, with the highest percentage of correct answers for the Group B, regarding the ability to perform a self-examination, the surgical removal of the lesion as the primary therapeutic method (which may, or may not be supplemented by radiotherapy and/or chemotherapy), the professional responsible for the treatment (medical oncologist) and, finally, the profile of an individual with the lesion (Caucasian man, 50 years old, smoker and drinker).

In this context, a significant portion (56.0%) of Group A declared that only the dentist was able to examine the oral cavity. About 61.0% of the first-semester students and 29.0% of the seventh-semester students indicated the dentist as the professional responsible for the treatment of the disease.

**Figure 1:**
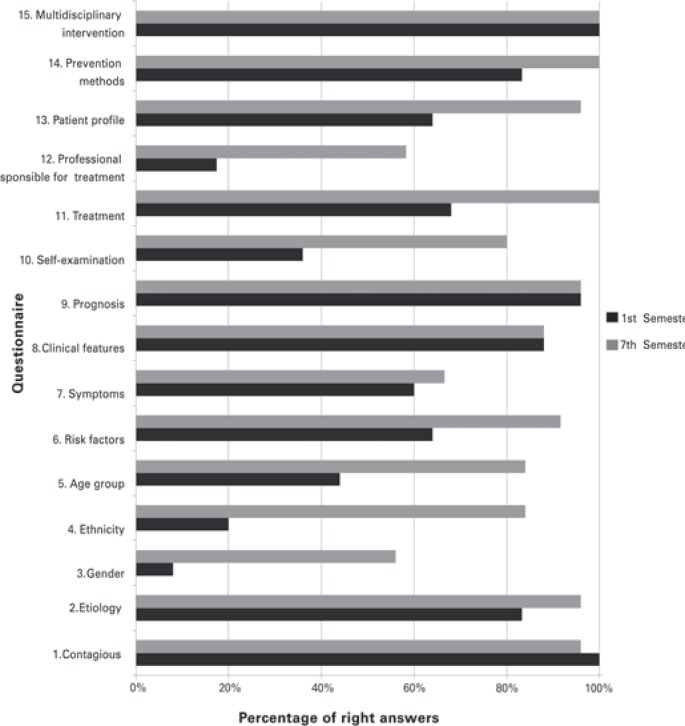
Number of right answers to 15 questions given to first and seventh semester undergraduate dental students

For a complete assessment of the results, the correct answers percentages of the first-semester and seventh-semester undergraduate students, referring to the 15 questions contained in the questionnaire, can be seen in figure 1.

To compare the frequencies of correct answers for the 15 questions of the questionnaire in each group and see the variables with the lowest percentage of correct answers, by applying Cochran's G test, tables 2 and 3 were prepared, respectively, for Groups A and B. The lines represent the students' answers, 1 for correct and zero for incorrect. The absence of a digit indicates that the question was dismissed because the respondent indicated more than one alternative.

**Table 2:** First-semester undergraduate dental students, as per correct (1) or incorrect answers (0) given to the questions

Students	Questions														
	1	2	3	4	5	6	7	8	9	10	11	12	13	14	15
1	1		1	0	0	0	0	1	1	0	1	0	1	0	1
2	1	0	0	0	0	0	0	1	1	0	1	0	0	1	1
3	1	1	0	0	0	1	1	0	1	0	0	1	1	1	1
4	1	1	0	0	0	1	1	0	1	0	0	1	1	1	1
5	1	1	0	0	0	1	1	0	1	0	0	1	0	1	1
6	1	1	0	0	0	1	1	1	1	0	1	0	0	1	1
7	1	1	0	0	0	1	1	1	1	1	1	0	1	1	1
8	1	1	0	0	0	0	0	1	1	0	1	0	0	0	1
9	1	1	0	0	1	0	0	1	1	1	0	0	1	1	1
10	1	1	0	0	0	0	1	1	1	0	1	0	0	1	1
11	1	1	0	0	1	0	1	1	1	1	1	0	1	1	1
12	1	1	0	0	1	1	1	1	1	0	1	0	1	1	1
13	1	1	0	0	1	1	1	1	1	0	1	0	1	1	1
14	1	1	0	0	1	1	1	1	1	0	1		1	1	1
15	1	1	0	0	1	1	1	1	1	1	1	0	0	1	1
16	1	1	0	0	1	1	0	1	1	0	0	0	1	1	1
17	1	1	1	0	0	1	0	1	1	1	1	0	1	1	1
18	1	1	0	0	0	1	1	1	1	1	1	1	1	1	1
19	1	1	0	0	0	0	0	1	1	0	1	0	0	0	1
20	1	0	0	1	1	1	1	1	1	0	0	0	1	1	1
21	1	1	0	1	0	1	1	1	1	1	1	0	0	1	1
22	1	0	0	1	1	1	1	1	1	0	1	0	1	1	1
23	1	1	0	1	1	0	0	1	1	1	0		0		1
24	1	1	0	1	0	0	0	1	1	0	0	0	1	0	1
25	1	0	0	0	1	1	0	1	0	1	1	0	1	1	1
Total of (1)	25	20	2	5	11	16	15	22	24	9	17	4	16	20	25
% of (1)	100.0	83.3	8.0	20.0	44.0	64.0	60.0	88.0	96.0	36.0	68.0	17.4	64.0	83.3	100.00

Cochran G test/G: 138.92 (p=0.0000).

**Table 3:** First-semester undergraduate dental students, as per correct (1) or incorrect answers (0) given to the questions

Students	Questions														
	1	2	3	4	5	6	7	8	9	10	11	12	13	14	15
1	1	1	0	1	1	1	0	1	0	1	1	1	1	1	1
2	1	1	1	1	1	1	1	1	1	1	1	1	1	1	1
3	1	1	1	1	0	1	1	1	1	1	1	0	1	1	1
4	1	1	1	1	1	1	1	0	1	1	1	1	1	1	1
5	1	1	1	1	1	1	1	1	1	1	1	1	1	1	1
6	1	1	1	1	1	1	1	1	1	1	1	1	1	1	1
7	1	1	1	1	1	1	0	1	1	1	1	0	1	1	1
8	1	1	1	0	1	1		1	1	1	1	1	1	1	1
9	1	1	0	1	1	1	1	1	1	1	1	0	1	1	1
10	1	1	1	1	1	1	0	1	1	0	1	0	1	1	1
11	1	1	1	1	1	1	0	1	1	1	1	1	1	1	1
12	1	1	0	0	0	0	1	1	1	0	1	0	1	1	1
13	1	1	0	1	0	1	1	1	1	1	1	0	1	1	1
14	1	1	0	1	1	1	1	1	1	1	1		1	1	1
15	1	1	0	0	1	1	1	1	1	1	1	1	1	1	1
16	1	1	0	1	0		0	1	1	1	1	0	0	1	1
17	1	1	0	1	1	1	1	1	1	0	1	1	1	1	1
18	1	1	0	1	1	1	1	0	1	1	1	0	1	1	1
19	1	1	0	0	1	1	0	1	1	1	1	1	1	1	1
20	1	1	1	1	1	1	0	1	1	1	1	1	1	1	1
21	1	1	0	1	1	1	1	0	1	0	1	0	1	1	1
22	1	1	1	1	1	1	1	1	1	1	1	1	1	1	1
23	0	0	1	1	1	0	1	1	1	0	1	0	1	1	1
24	1	1	1	1	1	1	1	1	1	1	1	1	1	1	1
25	1	1	1	1	1	1	0	1	1	1	1	1	1	1	1
Total of (1)	24	24	14	21	21	22	16	22	24	20	25	14	24	25	25
% of (1)	96.0	96.0	56.0	84.0	84.0	91.6	66.6	88.0	96.0	80.0	100.0	58.3	96.0	100.0	100.0

Cochran G test/G: 70.32 (p=0.0000).

Concerning the first-semester students, the percentages of correct answers to the questions related to gender, ethnicity, age, risk factors, symptoms, self-examination, treatment, professional responsible for the treatment and patient with the profile of the disease were significantly lower than for the remaining questions, and the calculated G value was 138.92 (p=0.0000). In turn, for the seventh-semester students, the questions with the lowest percentages of correct answers were those related to gender, symptoms and professional responsible for the treatment. For this group, the calculated G value was 70.32 (p=0.0000).

Subsequently, the Kendall's concordance test was applied in order to analyze a possible concordance between the frequency of correct answers of the students of both semesters. Table 4 shows the percentage of correct answers to the questions contained in the questionnaire. Frequencies were ordered in sequence and in ascending order of percentage of correct answers.

**Table 4:** Groups A and B of dentistry course, as per questions asked, organized in crescent order of right answers and their ranking

Questions	1^st^ Semester		7^th^ Semester	
	Right answers	Rank	Right answers	Rank
Contagious	25	14.5^th^	24	10.5^th^
Etiology	20	10.5^th^	24	10.5^th^
Prevalent gender	2	1^st^	14	1.5^th^
Prevalent ethnicity	5	3^rd^	21	5.5^th^
Age group	11	5^th^	21	5.5^th^
Risk factors	16	7.5^th^	22	7.5^th^
Symptoms	15	6^th^	16	3^rd^
Clinical features	22	12^th^	22	7.5^th^
Prognosis	24	13^th^	24	10.5^th^
Self-examination	9	4^th^	20	4^th^
Treatment	17	9^th^	25	14^th^
Professional responsible for treatment	4	2^th^	14	1.5^th^
Patient profile	16	7.5^th^	24	10.5^th^
Prevention methods	20	10.5^th^	25	14^th^
Multidisciplinary intervention	25	14.5^th^	25	14^th^

Kendall's concordance test. W coefficient concordance of 0.90 (p=0.0315).

The W coefficient of 0.90 (p=0.0315) shows a great and significant concordance in the order of correct answer percentages observed among students of Groups A and B. Although the percentages of correct and wrong questions have varied in the groups, the students, in general, were less successful in the same variables.

## DISCUSSION

In accordance with INCA data, Brazil has high incidence and prevalence rates of oral cancer, which ranks fifth as most frequent types of cancer in men, and twelfth as most frequent cancer in women (excluding non-melanoma skin cancer).^(4)^

Undoubtedly, health promotion efforts are essential. To effectively help prevent a disease it is important to know how to identify the risk population and the risk factors involved and then establish measures that are beneficial and favorable to the individuals concerned, in order to reduce the likelihood of its occurrence. From these premises, it is vitally important to assess the knowledge of students and health professionals, through qualitative research and other evaluation tools, because they, in particular, must be prepared and competent to work in community service.^(8,9,10,11)^

In this context, the observed recurrence of correct answers to questions related to epidemiological characteristics was statistically significant. However, even for the seventh-semester undergraduate students, knowledge of the prevalent patient sex proved to be significantly lower compared to the other variables evaluated, since about little over half of the respondents (56.0%) correctly answered male as the sex most frequently affected by the lesion. These samples agree with the results found in a previous study, in which about 52.0% of the respondents, comprised of dentistry and nursing students, answered correctly.^(12)^

With regard to risk factors, 64.0% in Group A and 91.6% of Group B indicated smoking as the most important factor, with a significant difference between the response rates. These results are higher than those found in other studies that evaluated the general population and groups not sensitized by prevention campaigns.^(3,9)^ However, in studies conducted among students and health care professionals, smoking was widely indicated as one of the main risk factors, and the percentage of correct answers ranged between 83.0 and 100.0%, in agreement with the results presented by the seventh-semester undergraduate students.^(1,6,7,13,14)^

Whereas smoking, alcoholism, chronic solar radiation (for lip cancer), and human papilloma virus (HPV) are identified as the main risk factors for oral cancer, the students were questioned only about smoking, in order to evaluate the specific knowledge of this factor, for it is highly associated with the development of cancer. Besides, it was not the objective of the research to highlight all factors, to avoid inducing the students to choose the alternative that would address the risks more broadly.

Regarding pain symptoms, 60.0% of students in Group A and 66.66% of students in Group B considered them present in the advanced stages of the disease. These results are similar to those presented by other authors, whose percentage of responses ranged from 42.8 to 86.8%.^(1,2,8)^

Similarly, there was a statistically significant difference in the responses of students, when asked about oral self-examination: 80.0% of Group B and 36.0% of Group A answered that it was possible to do it. The literature shows that 39.2% of third-year high school students claimed to have knowledge of the oral self-assessment.^(15)^ Raising awareness of the self-examination is important, because it contributes to the encouragement of an early diagnosis, enabling the individual to seek appropriate treatment in the early stages of the disease, which improves the prognosis (according to TNM classification).^(16)^

There was little difference regarding knowledge and practices about oral cancer among students of fourth to tenth semesters of graduation.^(17)^ In another study, the level of knowledge of third and fifth year dentistry students was better than those of the first-year students.^(18)^ In the present study, there was a significant concordance in the answer frequency order observed among students of the first and seventh semesters. Although the correct and wrong answer percentages have varied in groups, the students were less successful, in general, on the same variables. It is possible to infer that a specific approach is necessary, mainly aimed at raising awareness of the prevalent gender, the professional responsible for the treatment, the symptoms, and the self-examination of oral cancer.

Therefore, in the aforementioned studies, we found that the percentages of correct answers depended on the evaluated population. Healthcare professionals and seventh-semester undergraduate students have shown good knowledge of risk factors, clinical aspects, and prevention methods.^(6,7,13,19)^ On the other hand, studies conducted among the population showed that the knowledge of these factors was lower.^(3,9)^

On the other hand, even among professionals, few could identify the most common sites of disease development; a small percentage of them was prepared to perform biopsy procedures; and less than 5.0% knew the proper procedures for the examination of the tongue, showing a clear deficiency in this regard.^(2,6,7)^ The detailed examination of oral structures is essential because it can show early signs of change, favoring an early diagnosis.^(2,6,7)^

## CONCLUSION

Concerning the variables related to epidemiological characteristics, risk factors, self-examination, therapeutic solutions and profile of an individual with the lesion, the seventh-semester undergraduate students were significantly more likely to correctly answer the questions.

The questions about prevalent sex, pain symptoms and professional responsible for the treatment revealed that the students of the seventh semester had poor knowledge of oral cancer, with significantly lower percentages of correct answers than in the other questions.

In short, there is a significant concordance in the percentages of correct and wrong answers in the samples of first-semester and seventh-semester students, which requires a specific approach directed to the deficiencies presented by these future professionals.

## Appendix 1 Questionnaire

Answer ALL the questions below by checking ONLY ONE alternative.

1. Oral cancer:
  - Is not a disease.
  - Is a contagious disease.
  - Is a disease, but is not contagious.
2. Cancer occurs due to:
  - Uncontrolled cell growth.
  - Cell death.
  - Entry of bacterias into cells.
3. Oral cancer occurs more frequently:
  - In men.
  - In women.
  - Same in both genders.
4. Among the ethnic groups below, which is the most influenced in developing lip and face cancer?
  - Black.
  - Caucasian.
  - Any of the above.
5. At what age most cases of mouth cancer are diagnosed?
  - Under 20 years.
  - Between 20 and 40 years.
  - Over 40 years.
6. What is the main risk factor for oral cancer?
  - Smoking.
  - Genetic inheritance.
  - There is no established risk factor for this disease.
7. Oral cancer:
  - Causes pain.
  - Causes pain at advanced stages of the disease.
  - Causes pain at all stages of the disease.
8. Can an ulcer on the tongue that does not heal for a period of more than 15 days indicate risk of oral cancer?
  - No, because oral cancer does not appear as an ulcer.
  - Yes, it is necessary to consult a dental surgeon.
  - Because 15 days is too short a period to indicate a serious lesion.
9. Oral cancer:
  - Certainly has no cure.
  - Has a cure, regardless of when the diagnosis is made.
  - The earlier the diagnosis, the greater the chance of cure.
10. Is it possible to perform an oral cancer self-examination?
  - No, only a dental surgeon can do it.
  - It depends. You need someone else to help you.
  - Yes, you can perform a self-examination.
11. The treament of oral cancer is mainly based on:
  - There is no treatment.
  - Medications.
  - Surgery.
12. The following professional is responsible for the treatment:
  - Physician.
  - Dental surgeon.
  - Physician or dental surgeon, no specific professional.
13. Of the options below, which most closely matches the profile of a person with oral cancer?
  - Caucasian man, 50 years old, smoker and drinker.
  - Black woman, 20 years old, family history of oral cancer.
  - Black man, 20 year old, sunlight exposure with no protection.
14. How can oral cancer be prevented:
  - By taking specific medications for prevention.
  - Not smoking, avoiding alcohol and using sunscreen.
  - You can not prevent this cancer because it is hereditary.
15. The prevention of oral cancer depends on:
  - Only health care professionals, for they are prepared to do this.
  - Everyone can contribute. It is necessary to raise awareness of the risk factors in order to avoid them.
  - There is no way to prevent this cancer because people are predetermined to have it, and there is no way to change this.
